# Supplementary material for: Prospection of Peptide Inhibitors of Thrombin from Diverse Origins Using a Machine Learning Pipeline
Source: Bioengineering (Basel). 2023 Nov 9;10(11):1300. doi: 10.3390/bioengineering10111300 (PMC10669389; doi:10.3390/bioengineering10111300)
Supplement: Supplementary file 1 [file bioengineering-10-01300-s001.zip › bioengineering-2637560-supplementary.docx]

**SUPPLEMENTARY INFORMATION**


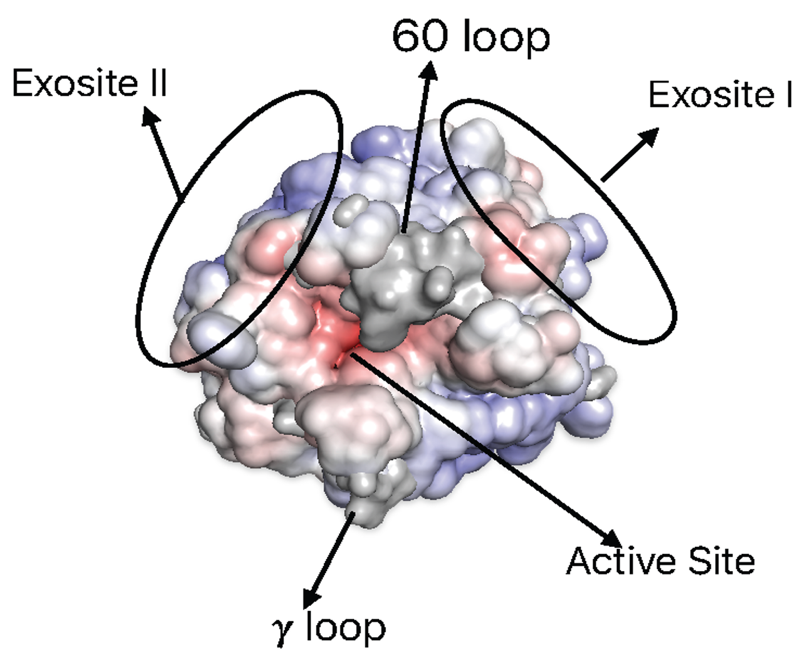


**Figure S1.** Thrombin geography (1PPB) in standard orientation.


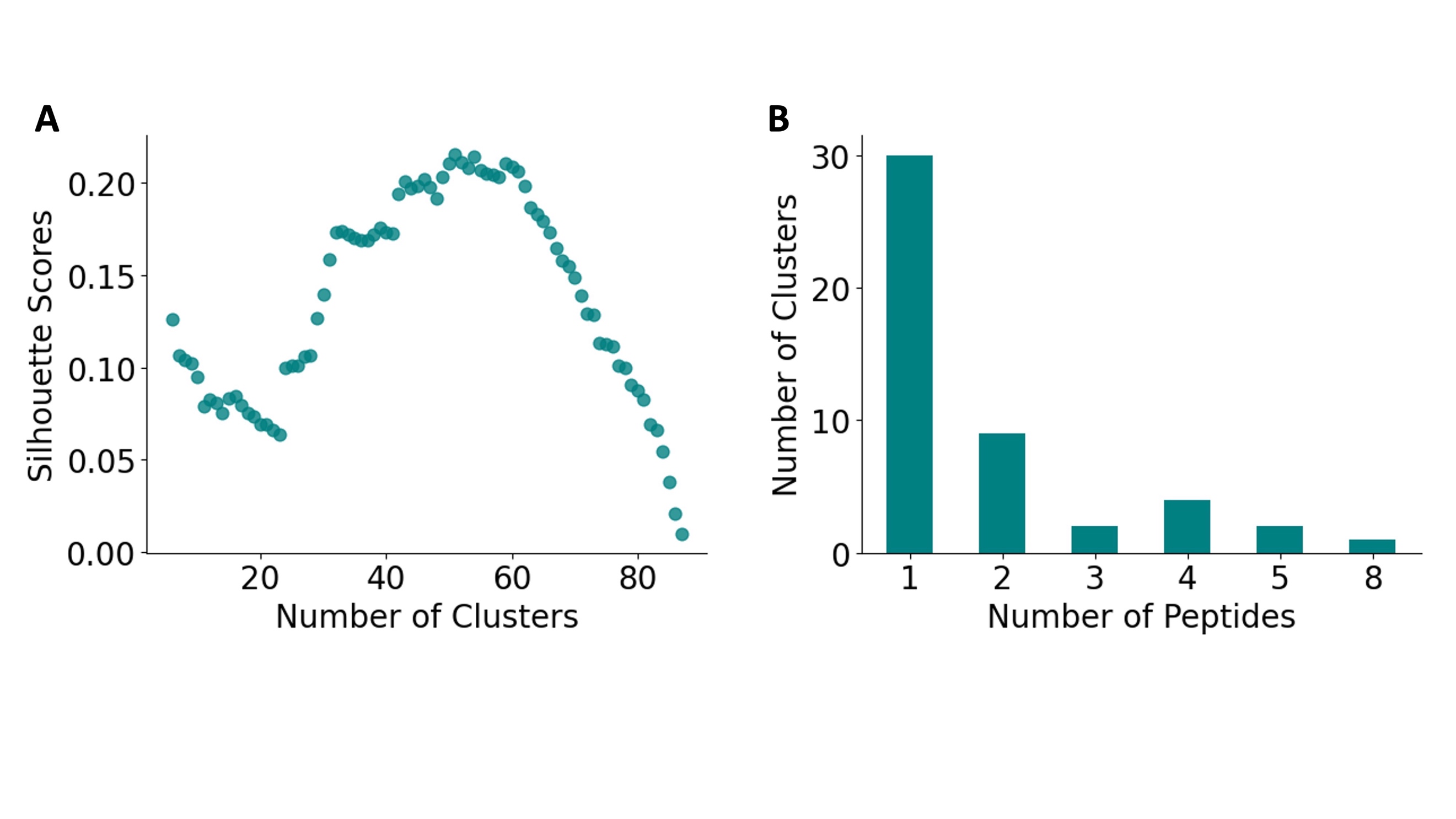


**Figure S2.** Agglomerative clustering of peptides in positive dataset. (A) A maximum in silhouette scores correspond to an optimum number of clusters; (B) number of peptides per cluster.


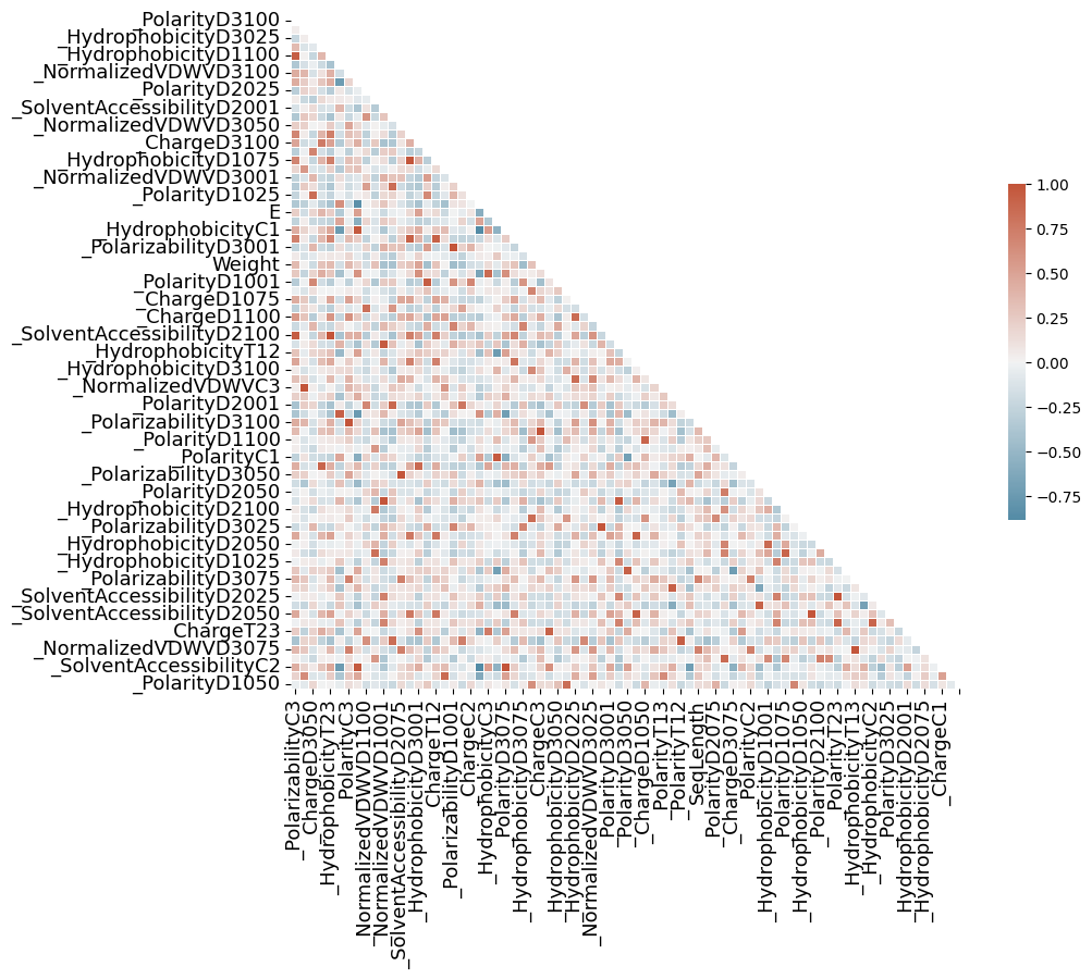


**Figure S3.** Highly correlated (>80%) features in the positive dataset.


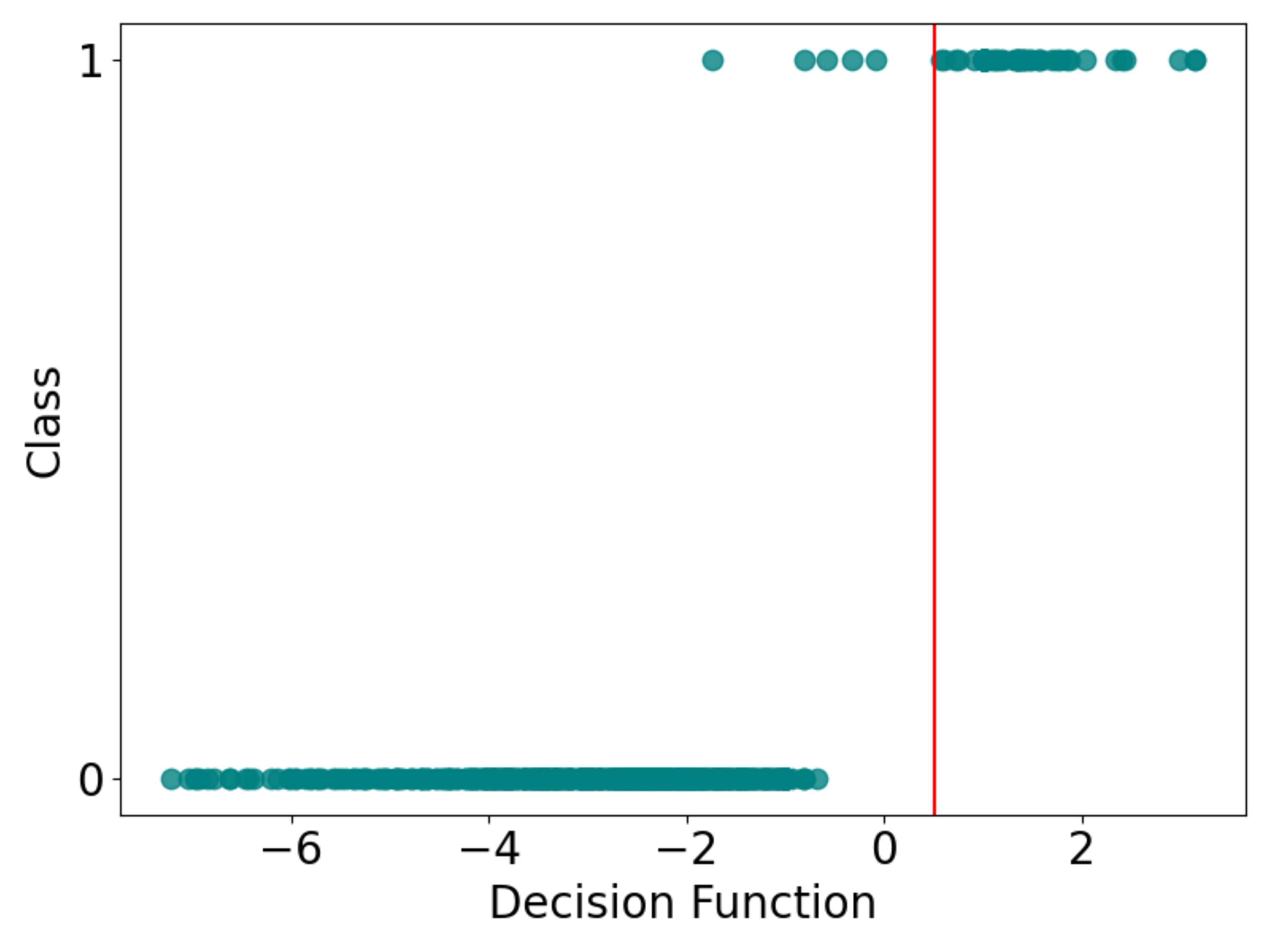


**Figure S4.** Decision function of peptides in the positive (1) and negative (0) datasets, as estimated by feature-reduced SVM-Linear classification model. A decision function of 0.5 (red line) eliminates all false positives.


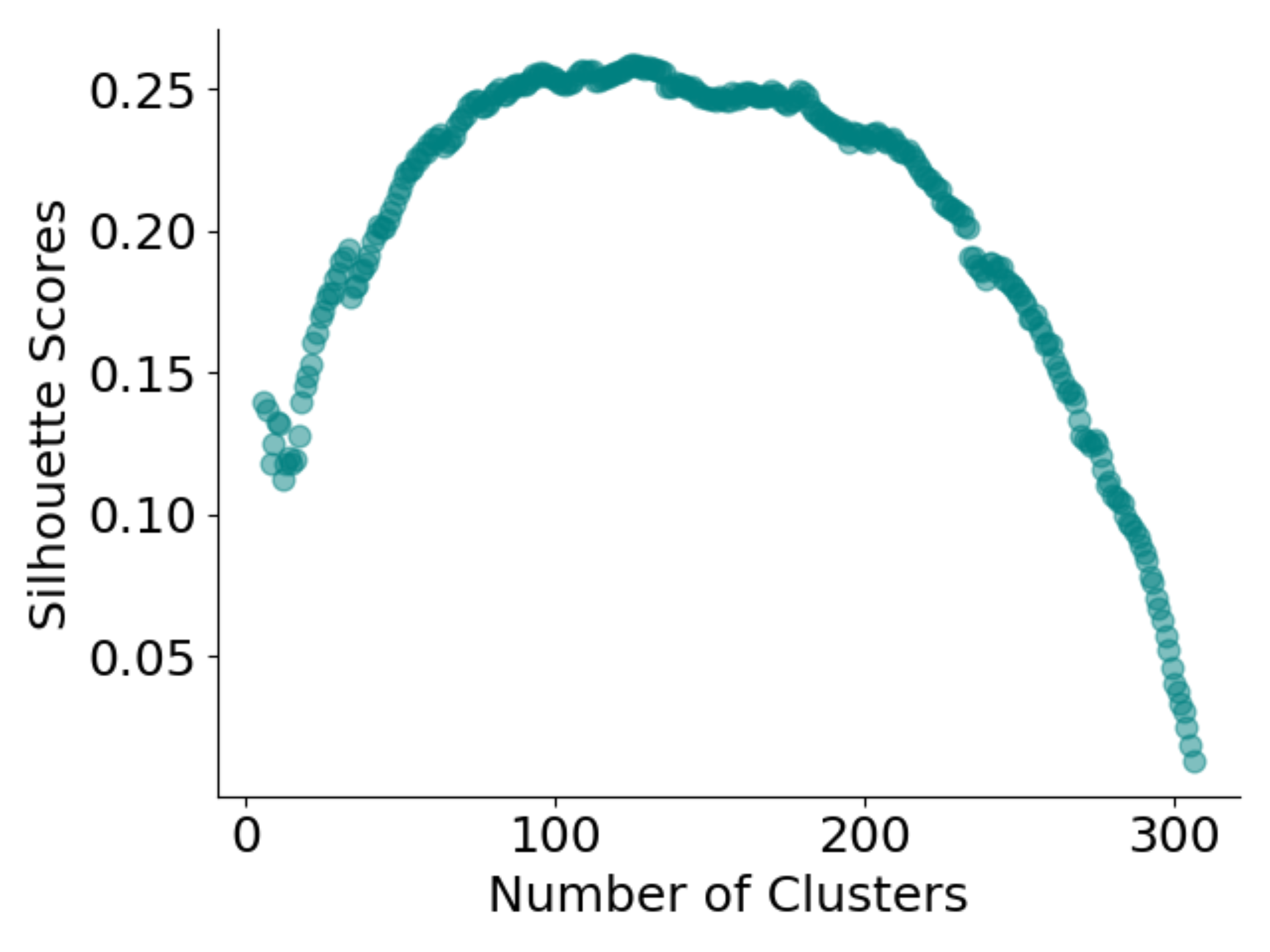


**Figure S5.** Agglomerative clustering of peptide hits.

**
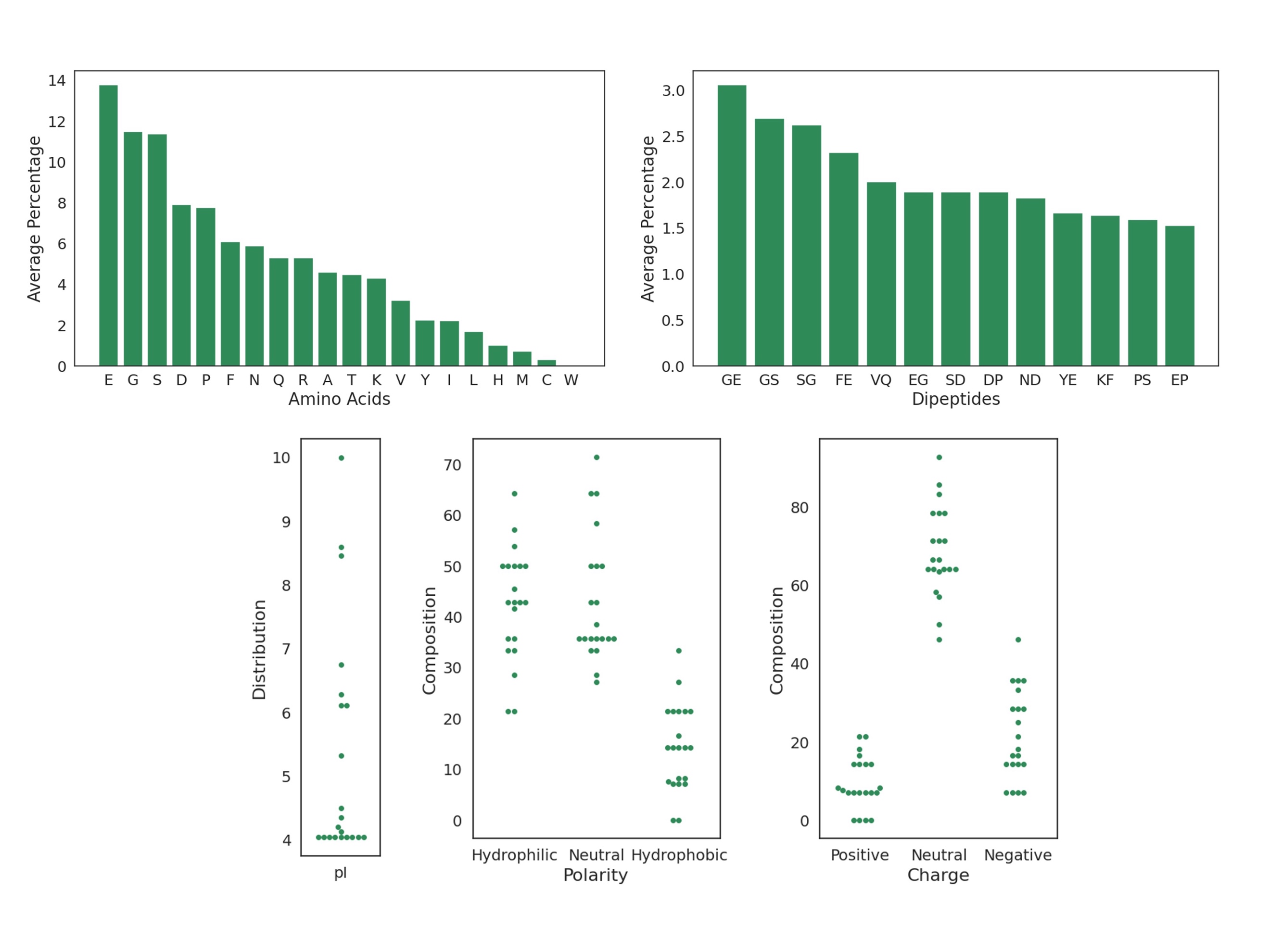
**

**Figure S6.** Characteristics of peptide hits*.*

| **Name** | **Thrombin–peptide complex** | **Binding Site** | **Binding Residues** |
| --- | --- | --- | --- |
| P11 (Hirugen) |  | Exosite 1 | E23, I24, K36, S37, P38, G69, K70, H71, S72, T74, R75, W141, N143, K145, G150, Q151, P152, S153, V154, L155, Q156, V157 |
| P37 (Avathrin) |  | Active site and Exosite 1 | P38, Q39, E40, L40, K70, H71, S72, R73, T74, Y76, E77, R77a, R97, W141, L144, E146, K149, G150, Q151, P152, S153, L154, E192 |

**Figure S7.** Confirmation of binding sites of two positive peptides on thrombin using CABS DOCK. Thrombin structures are rotated from standard orientation for better visualization of the binding sites. The peptides are colored in red and the binding locations within 5 Å distance are colored in blue.


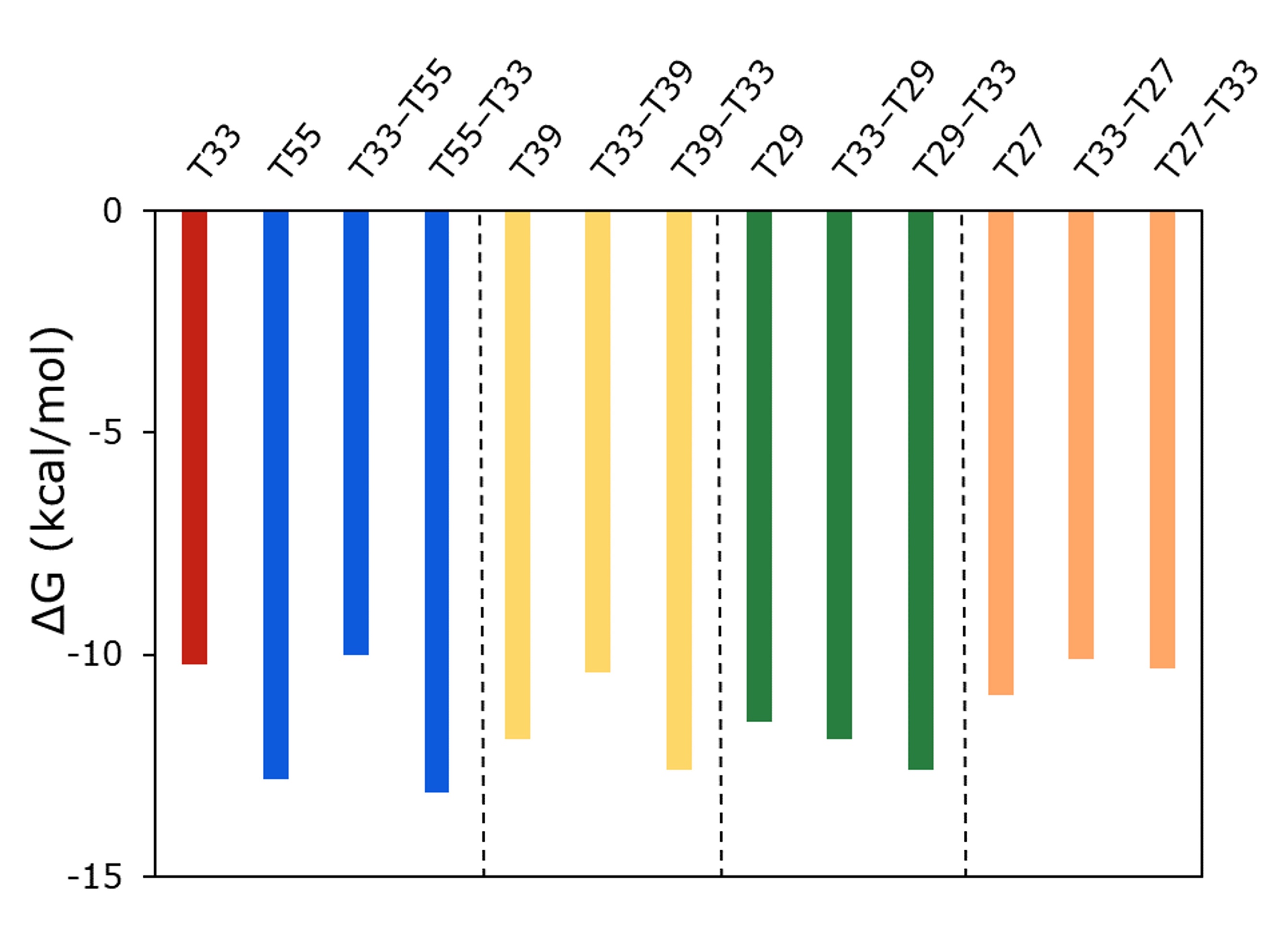


**Figure S8.** Design of a bivalent peptide. (A) Free energy change in binding of thrombin to bivalent peptide composed of T33 peptide combined with either T55, T39, or T27 peptides in either N-C or C-N concatenation.

**Table S1.** Positive peptides.

| **Name** | **Sequence** | ***K_I_* (nM)** | **Source** | **Reference** |
| --- | --- | --- | --- | --- |
| P1 | FPR | 61000 | Synthetic | [50] |
| P2 | RPPGF | 1750000 | Synthetic | [51] |
| P3 | NAESLRK |  | *Crassostrea gigas*  (P-3-CG) | [52] |
| P4 | AGFAGDDAPR |  | *Tenebrio molitor* larvae | [53] |
| P5 | SLVDAIGMGP |  | *Tenebrio molitor* larvae | [53] |
| P6 | SDQGDVAEPK |  | Isolated | [54] |
| P7 | TKLTEEEKNR |  | Milk peptide | [55] |
| P8 | DFEAIPEEYL |  | Synthetic | [54] |
| P9 | DFEEIPEEYL |  | Synthetic | [54] |
| P10 | IEELEEELEAER |  | *Crassostrea gigas*  (P-2-CG) | [56] |
| P11 | NGDFEEIPEEYL | 500000 | C-terminal of hirudin | [57] |
| P12 | KNAENELGEVTVR |  | *Mytilus edulis* mussels  (peptide 26) | [58] |
| P13 | NGDFEEIPEEYLQ |  | Synthetic | [59] |
| P14 | AEVEYEALYPEDD |  | Isolated | [54] |
| P15 | EDDNPGPPRACPGE |  | Isolated | [54] |
| P16 | AEVEYEALYPEDDL |  | Isolated | [59] |
| P17 | DNEEGFFSARGHRPL |  | Human fibrinogen | [60] |
| P18 | DFLAEGGGVRGPRVV |  | Human fibrinogen | [60] |
| P19 | VVYTDCTESGQNLCLCE |  | *Hirudo medicinalis* | [61] |
| P20 | FPRPGGNGDFEEIPEEYL | 64.5 | Synthetic | [62] |
| P21 | MHKTAPPFDFEAIPEEYL |  | Isolated | [54] |
| P22 | ISKQGLGGDFEEIPSDEIIE | 5.76 | *Amblyomma variegatum*  (avathrin) | [40] |
| P23 | FPRPGGGGNGDFEEIPEEYL | 156 | Synthetic | [62] |
| P24 | VRPEHPAEVEYEALYPEDDL |  | Isolated | [59] |
| P25 | VRPEHPAETEYESLYPEDDL |  | Isolated | [59] |
| P26 | WDPRPQLHNDGDFEPIPEEYLQ | 39 | Synthetic | [63] |
| P27 | LDPRPQSHNDGDFEEIPEEYLQ | 260 | Synthetic | [63] |
| P28 | GSAVPRPQIHNDGDFEEIPEEYLQ | 41 | Synthetic | [63] |
| P29 | QTAVPKISKQGLGGDFEEIPSDEIIE | 0.835 | *Amblyomma variegatum* | [40] |
| P30 | SDQADRAQPKLHRNAPQGDFEAIPDEYL |  | Isolated | [54] |
| P31 | SDQSGRAQPKLPRNAPQGDFEAIPDEYL |  | Isolated | [54] |
| P32 | SDQGDVAEPKMHKTAPPFDFEAIPEEYL |  | Isolated | [54] |
| P33 | SGGHQTAVPKIHKQGLGGDFEEIPSDEII | 1.23 | *Amblyomma variegatum* | [40] |
| P34 | SDQADVAEPKMHKTAPPGDFEAIPEEYLD |  | Isolated | [54] |
| P35 | EDYAAIEASLSETFNTAADPGRRLGEGSKP | 0.304 | *Anopheles albimanus* | [64] |
| P36 | SGGHQTAVPRISKQGLGGDFEEIPSDEIIE | 0.172 | *Amblyomma variegatum* | [40] |
| P37 | SGGHQTAVPKISKQGLGGDFEEIPSDEIIE | 0.545 | *Amblyomma variegatum* | [40] |
| P38 | SDGHDTAVPKISKQGLGGDFEEIPSDEIIE | 0.932 | *Amblyomma variegatum* | [40] |
| P39 | SGGHQTAVPKIAKQGLGGDFEEIPSDEIIE | 6.075 | *Amblyomma variegatum* | [40] |
| P40 | SGGHQTAVPAISKQGLGGDFEEIPSDEIIE | 32.04 | *Amblyomma variegatum* | [40] |
| P41 | GEPGAPIDYDEYGDSSEEVGGTPLHEIPGIRL | 150 | *Glossina morsitans*  *morsitans* | [65] |
| P42 | SDQGDVAEPKMHKTAPPFDFEAIPEEYLDDES | 0.0104 | *Amblyomma variegatum* | [66] |
| P43 | SDQGDVAEPKMHKTAPPFDFEAIPEEYLDDHS |  | Isolated | [54] |
| P44 | CPHAKHRVCGANGEVYDNECFLNKAGIEPAESW  ETCRGHE | 4000 | *Carcinoscorpius*  *rotundicauda* | [67] |
| P45 | VSYTDCTESGQNYCLCVGSNVCGEGKNCQLSSS  GNQCVHGEG |  | *Hirudinaria manillensis* | [68] |
| P46 | IDCPDKSKCKDDNTCCEVPSGKFACCDLPEAVC  CADKMHCCPPKS |  | *Hirudo nipponia*  (granulin-like) | [69] |
| P47 | AHPLCLLDPPFGFCQSSISRFAPVVGKCREYIYGG  CSGKANNFQAQAKCQANCG | 26000 | *Vespa bicolor*  (bicolin) | [70] |
| P48 | FQGNPCECPRALHRVCGSDGNTYSNPCMLTCAK  HEGNPDLVQVHEGPCDEHDHDF | 7.5 | *Dipetalogaster maximus*  (dipetalin I) | [71] |
| P49 | IRFGMGKVPCPDGEVGYTCDCGEKICLYGQSCN  DGQCSGDPKPSSEFEEFEIDEEEK | 0.0001 | *Haemadipsa sylvestris*  (haemadin) | [72] |
| P50 | YPERDSANRGSQEKERALLVKVQERSSQDDYDE  YDADETTLSPDPDAPTARPRLGRKNA | 290 | *Haemaphysalis bispinosa* | [73], [74] |
| P51 | YPERDSAKEGNKGQKRARLVNVQERSGETDYD  EYEENENTPTPDPSAPTARPRLGRKNA | 210 | *Haemaphysalis bispinosa* | [73], [74] |
| P52 | KPNLQSRSDDGVDESDYDTYPDDNNDDSGERN  GGSEPAKPRLPVPGSGRDSERIPVPVD | 0.4 | *Hyalomma marigatum* | [75] |
| P53 | YPERDSAKEGNQEQERALHVKVQKRTDGDADY  DEYEEDGTTPTPDPTAPTAKPRLRGNKP | 55.6 | *Haemaphysalis longicornis*  (unmodified madanin-1) | [76] |
| P54 | YPERDSAKDGNQEKERALLVKVQERYQGNQGD  YDEYDQDETTPPPDPTAQTARPRLRQNQD |  | *Haemaphysalis longicornis*  (madanin-2) | [77] |
| P55 | APQYAPGDEPSYDEDTDDSDKLVENDTSITDEDY  AAIEASLSETFNTAADPGRRLGEGSKP | 0.034 | *Anopheles albimanus* | [64] |
| P56 | APQYAPGDEPSYDEDTDDSDKLVENDTSITDEDY  AAIEASLSETFNTAADPGRNLGEGSKP | 1.09 | *Anopheles albimanus* | [64] |
| P57 | APQYAPGDEPSYDEDTDDSDKLVENDTSITDEDY  AAIEASLSETFNTAAEPGRRLGEGSKP | 44.75 | *Anopheles albimanus* | [64] |
| P58 | APQYAPGDEPSYDEDTDDSDKLVENDTSITDEDY  AAIEASLSETFNTAAAPGRRLGEGSKP | 203.6 | *Anopheles albimanus* | [64] |
| P59 | VSYTDCTSGQNYCLCGGNFCGDGKHCEMDSEN  KCVDGEGTPKRQTSGPSDFEEFSLDDIEQ | 0.0078 | *Hirudinaria manillensis*  (hirullin P18) | [78] |
| P60 | MRYTACTESGQNQCICEGNDVCGQGRNCQFDSS  GKKCVEGEGTRKPQNEGQHDFDPIPEEYLS | 0.000058 | *Hirudinaria manillensis* | [79] |
| P61 | VSYTDCTESGQNYCLCVGSNVCGEGKNCQLSSS  GNQCVHGEGTPKPKSQTEGDFEEIPDEDILN |  | *Hirudinaria manillensis*  (HM2) | [80] |
| P62 | VSYTDCTESGQNYCLCVGGNLCGGGKHCEMDG  SGNKCVDGEGTPKPKSQTEGDFEEIPDEDILN |  | *Hirudinaria manillensis* | [68] |
| P63 | ITYTDCTESGQNLCLCEGSNVCGKGNKCILGSNG  KGNQCVTGEGTPNPESHNNGDFEEIPEEYLQ | 0.0002 | Recombinant hirudin | [81] |
| P64 | ITYTDCTESGQNLCLCEGSNVCGNGNKCKLGSD  GEENQCVTGEGTPKPQSHNDGDFEEIPEEYLQ |  | *Hirudo medicinalis* | [61] |
| P65 | VVYTDCTESGQNLCLCEGSNVCGQGNKCILGSD  GEKNQCVTGEGTPKPQSHNDGDFEEIPEEYLQ | 0.000022 | *Hirudinaria manillensis* | [82] |
| P66 | LTYTDCTESGQNLCLCEGSNVCGQGNKCILGSD  GEKNQCVTGEGTPKPQSHNDGDFEEIPEEYLQ | 0.00006 | Lepirudin (LT-desulfato  hirudin) | [83] |
| P67 | ERGVCACPRIYMPVCGSNLKTYNNDCLLRCEINS  DLGRANNLRKIADQACDNLTDNVNDFIPQEY | 320 | *Aedes aegypti* | [84] |
| P68 | ECENTECPRACPGEYEFDEDGCNTCVCKGCDDA  QCRCSSDANGCESFCTCNTRCSAADECNPRCTCK | 0.000012 | *Theromyzon tessulatum*  (threomin) | [85] |
| P69 | NRYSVCTETGQNLCLCEGSDLCSLDNHCEIGSNG  KNRCVKGEGKPKKPQSNSDLPEEKYEPIPIEDYDK | 2970 | *Hirudo nipponia*  (hirudin-HN) | [86] |
| P70 | QPKEKTKGVEVEGNPATLISARQMDVSYDEYED  NGPDVIPGEPAKPRGGPKNGAASGKFDQIPDFSSESH | 214.4 | *Haemaphysalis longicornis* (chimadinin) | [87] |
| P71 | SAGPITLQLDDDDDDDSGIPIFEMDDEDEDSNDN  QKFPLSFERFPENEKNQVGLRARFNKFMAKFTSL  FGRRRGVNVPNAA |  | *Haematobia irritans*  (thrombostatin) | [88] |
| P72 | APQYARGDVPTYDEEDFDEESLKPHSSSSSDDGE  EEFDPSLLEEHADAPTARDPGRNPEFLRNSNTDE  QASAPAASSSESDE | 0.0035 | *Anopheles gambiae* (cE5) | [89] |
| P73 | LEENDCACPRVLHRVCGSDGNTYSNPCTLDCAK  HEGKPDLVQVHEGPCDPNDHDFEDPECDNKFEP  VCGTDHITYSNLCHLECAAFTTSPGVEVKYEGEC  HAE | 0.025 | *Triatoma infestans*  (infestin-1) | [90] |
| P74 | EGGEPCACPHALHRVCGSDGETYSNPCTLNCAK  FNGKPELVKVHDGPCEPDEDEDVCQECDGDEYK  PVCGSDDITYDNNCRLECASISSSPGVELKHEGPC  RT | 0.0002 | *Rhodnius prolixus*  (rhodinin) | [91] |
| P75 | FQGNPCECPRALHRVCGSDGNTYSNPCMLTCAK  HEGNPDLVQVHEGPCDEHDHDFEDTCQCDDTFQ  PVCDDEITYRNLCHLECATFTTSPGVEVKHGECH  PETKVN | 0.078 | *Dipetalogaster maximus*  (dipetalin I+II) | [71] |
| P76 | FQGNPCECPRALHRVCGSDGNTYSNPCMLTCAK  HEGNPDLVQVHEGPCDEHDHDFEDTCQCDDTFQ  PVCGDDEITYRNLCHLECATFTTSPGVEVKHEGE  CHPETKV | 0.0000493 | *Dipetalogaster maximus* (dipetalogastin) | [92] |
| P77 | LNVRCNNPHTANCENGAKLESYFREGETCVGSP  ACPGEGYATKEDCQKACFPGGGDHSTNVDSSCF  GQPPTSCETGAEVTYYDSGSRTCKVLQHGCPSSE  NAFDSEIECQVACGVSME | 0.00489 | *Ornithodoros savignyi*  (savignin) | [93] |
| P78 | LNVLCNNPHTADCNNDAQVDRYFREGTTCLMSP  ACTSEGYASQHECQQACFVGGEDHSSEMHSSCL  GDPPTSCAEGTDITYYDSDSKTCKVLAASCPSGE  NTFESEVECQVACGAPIEG | 0.001 | *Ornithodoros moubata*  (ornithodorin) | [94] |
| P79 | QRNGFCRLPAEPGICRAFMPRYYFDVEKGQCEQ  FIYGGCKGNENNFETLKECQDACGEPERASDFEK  ADFETGCKAAPETGLCKASFERWFFNAASGECE  EFIYGGCGGNDNNYENKEECEFACKY |  | *Haemaphysalis longicornis*  (hemalin) | [95] |
| P80 | DCPPDWSSYEGSCYRVFEQKMNWEDAEKFCTQ  QQTGGHLVSFQSSEEADFVVSLTSPILRDSFVWT  GLSDVWKGCRFEWSDGSDLSYKDNYQFVFSEY  ECVASKTKNNKWRIIPCTKLEYFVCEFQA |  | *Bothrops insularis* | [96] |
| P81 | QRNGFCRLPADEGICKALIPRFYFNTETGKCTMF  SYGGCGGNENNFETIEECQKACGAPERVNDFESA  DFKTGCEPAADSGSCAGQLERWFYNVQSGECET  FVYGGCGGNDNNYESEEECELVCKNM | 1.8 | *Boophilus microplus* | [97] |
| P82 | QRNQMCQQPRTQGSCDASNQITKFFYTGSGCTS  APVCSDTDGGYGTEDECIQACTVQGGHHNEGA  GEEGCSGDPPRGDCGGQVEERYYFDSTTRTCQT  FEYRGCSSGNPDNSYETEIECEIACPSASS | 0.007 | *Argas monolakensis*  (monobin) | [98] |
| P83 | DCPSDWSPYGQYCYKFFQQKMNWADAERFCSE  QAKGGHLVSFQSDGETDFVVNLVTEKIQSSDLYA  WIGLRVQNKEKQCSSKWSDGSSVSYENVVGRT  VKKCFALEKEQEFFVWINIYCGQQNPFVCKSPPP |  | *Bothrops insularis* | [96] |
| P84 | AEGDDCSIEKAMGDFKPEEFFNGTWYLAHGPGV  TSPAVCQKFTTSGSKGFTQIVEIGYNKFESNVKFQ  CNQVDNKNGEQYSFKCKSSDNTEFEADFTFISVS  YDNFALVCRSITFTSQPKEDDYLVLERTKSDTDP  DAKEIC | 0.003 | *Triatoma pallidipennis*  (triabin) | [99] |
| P85 | QRVPGYCKKKPAVGPCKALIEKWYFDYSTQSCK  TFYYGGCGGNGNKFSSRKKCREACLPKRPSVPV  CKQMPDPGFCRAYMPHWFFNSKSGYCEGFVYG  GCQGNDNRFKSCWYQCMKKCRTAREANRLCW  KLTKEFNKKFLRNVPTAKPLPPK | 20 | *Amblyomma hebraeum*  (amblin) | [100] |
| P86 | AADISQWAGPLCLQEVDEPPQHALRVDYAGVTV  DELGKVLTPTQVMNRPSSISWDGLDPGKLYTLV  LTDPDAPSRKDPKFREWHHFLVVNMKGNDISSG  TVLSDYVGSGPPSGTGLHRYVWLVYEQEQPLSC  DEPILSNKSGDNRGKFKVETFRKKYNLGAPVAG  TCYQAEWDDYVPKLYEQLSGK | 380 | Rat brain | [101] |
| P87 | EVQLVESGGGLVQPGGSLRLSCAASGFTISNSGI  HWVRQAPGKGLEWVGWIYPTGGATDYADSVK  GRFTISADTSKNTAYLQMNSLRAEDTAVYYCAR  FWWRSFDYWGQGTLVTVSSASTKGPSVFPLAPS  SKSTSGGTAALGCLVKDYFPEPVTVSWNSGALT  SGVHTFPAVLQSSGLYSLSSVVTVPSSSLGTQTYI  CNVNHKPSNTKVDKKVEPKSC |  | Human Fab  phage library | [102] |
| P88 | DCPSDWSSHEGHCYKFFQQKMNWADAERFCSE  QAKGGHLVSFQSDGETDFVVNLVTEKIQSTDLY  AWIGLRVQNKEKQCSSKWSDGSSVSYENVVGR  TVKKCFALEKEQEFFVWINIYCGQQNPFVCKSPP  PDCPPDWSSYEGSCYRVFEQKMNWEDAEKFCT  QQQTGGHLVSFQSSEEADFVVSLTSPILRDSFVW  TGLSDVWKEQSFEWSDGSDLSYKDNYQFVFSE  YECVASKTKNNKWRIIPCTKLEYFVCEFQA | 15 | *Bothrops jararaca*  (bothrojaracin) | [103] |

**Table S2.** Composition-Transition-Distribution (CTD) descriptor groups for the amino acids in three classes for each physico-chemical property (based on [30]).

|  | **Group 1** | **Group 2** | **Group 3** |
| --- | --- | --- | --- |
| **Hydrophobicity** | Polar | Neutral | Hydrophobicity |
|  | R, K, E, D, Q, N | G, A, S, T, P, H, Y | C, L, V, I, M, F, W |
| **Normalized van der Waals Volume** | 0–2.78 | 2.95–4.0 | 4.03–8.08 |
|  | G, A, S, T, P, D, C | N, V, E, Q, I, L | M, H, K, F, R, Y, W |
| **Polarity** | 4.9–6.2 | 8.0–9.2 | 10.4–13.0 |
|  | L, I, F, W, C, M, V, Y | P, A, T, G, S | H, Q, R, K, N, E, D |
| **Polarizability** | 0–0.108 | 0.128–0.186 | 0.219–0.409 |
|  | G, A, S, D, T | C, P, N, V, E, Q, I, L | K, M, H, F, R, Y, W |
| **Charge** | Positive | Neutral | Negative |
|  | K, R | A, N, C, Q, G, H, I, L, M, F, P, S, T, W, Y, V | D, E |
| **Secondary Structure** | Helix | Strand | Coil |
|  | E, A, L, M, Q, K, R, H | V, I, Y, C, W, F, T | G, N, P, S, D |
| **Solvent Accessibility** | Buried | Exposed | Intermediate |
|  | A, L, F, C, G, I, V, W | R, K, Q, E, N, D | M, S, P, T, H, Y |

**Table S3.** Hyperparameters of classification models obtained using RandomSearchCV.

| **Model** | **Hyperparameters** |
| --- | --- |
| SVM Linear | gamma = 2.936, break_ties = False, C = 1.2162 |
| SVM RBF | gamma = 0.093, break_ties = True, C = 3.3265000000000002 |
| Random Forest | penalty = 'l2', C = 6.159000000000001 |
| Logistic Regression | n_estimators = 120, max_features = 0.3, max_depth = 8, criterion = 'entropy', ccp_alpha = 0.0 |
| KNN | weights = 'distance', p = 2, n_neighbors = 6, leaf_size = 31 |
| XGBoost | subsample = 1, n_estimators = 490, max_depth = 3, lambda = 9, gamma = 0, alpha = 0.2 |

**Table S4.** Hyperparameters of RFE/SFS models obtained using RandomSearchCV.

| **Model** | **Hyperparameters** |
| --- | --- |
| SVM Linear | gamma = 0.924, break_ties = False, C = 3.8823 |
| SVM RBF | gamma = 0.385, break_ties = False, C = 1.5085 |
| Random Forest | penalty = 'l2', C = 8.46 |
| Logistic Regression | n_estimators = 380, max_features = 0.3, max_depth = 7, criterion = 'gini', ccp_alpha = 0.0 |
| KNN | weights = 'distance', p = 2, n_neighbors = 8, leaf_size = 25 |
| XGBoost | subsample = 1, n_estimators = 460, max_depth = 3, lambda = 9, gamma = 0, alpha = 1.0 |

**Table S5.** Prediction of inhibition constants for peptides in positive dataset.

| **Name** | ***K_I_* (nM)** | ***K_I_* (nM) Predicted** |
| --- | --- | --- |
| P1 | 61000 | 531520.15 |
| P2 | 1750000 | 21992538.95 |
| P3 |  | 187.15 |
| P4 |  | 0.75 |
| P5 |  | 26275.60 |
| P6 |  | 113.55 |
| P7 |  | 185.61 |
| P8 |  | 29270000 |
| P9 |  | 29270000 |
| P10 |  | 185.61 |
| P11 | 500000 | 5484593.11 |
| P12 |  | 3.95 |
| P13 |  | 2488807.26 |
| P14 |  | 0.00048 |
| P15 |  | 0.0096 |
| P16 |  | 0.00039 |
| P17 |  | 199564.25 |
| P18 |  | 8.35 |
| P19 |  | 1.74 |
| P20 | 64.5 | 485.03 |
| P21 |  | 1478.34 |
| P22 | 5.76 | 18.14 |
| P23 | 156 | 115.95 |
| P24 |  | 243.09 |
| P25 |  | 138.04 |
| P26 | 39 | 268.44 |
| P27 | 260 | 1444.77 |
| P28 | 41 | 283.60 |
| P29 | 0.835 | 3.76 |
| P30 |  | 3.74 |
| P31 |  | 216.42 |
| P32 |  | 424.50 |
| P33 | 1.23 | 3.27 |
| P34 |  | 0.03 |
| P35 | 0.304 | 0.69 |
| P36 | 0.172 | 0.65 |
| P37 | 0.545 | 2.52 |
| P38 | 0.932 | 4.25 |
| P39 | 6.075 | 19.28 |
| P40 | 32.04 | 121.93 |
| P41 | 150 | 701.27 |
| P42 | 0.0104 | 0.029 |
| P43 |  | 0.267 |
| P44 | 4000 | 25872.72 |
| P45 |  | 0.00011 |
| P46 |  | 185.61 |
| P47 | 26000 | 205943.39 |
| P48 | 7.5 | 43.04 |
| P49 | 0.0001 | 0.00012 |
| P50 | 290 | 1405.41 |
| P51 | 210 | 1736.87 |
| P52 | 0.4 | 1.66 |
| P53 | 55.6 | 224.76 |
| P54 |  | 197.58 |
| P55 | 0.034 | 2.12 |
| P56 | 1.09 | 2.86 |
| P57 | 44.75 | 312.70 |
| P58 | 203.6 | 330.73 |
| P59 | 0.0078 | 0.0211 |
| P60 | 0.000058 | 0.00009 |
| P61 |  | 0.00003 |
| P62 |  | 0.147 |
| P63 | 0.0002 | 0.0002 |
| P64 |  | 0.00086 |
| P65 | 0.000022 | 0.00002 |
| P66 | 0.00006 | 0.0001 |
| P67 | 320 | 1565.91 |
| P68 | 0.000012 | 0.00002 |
| P69 | 2970 | 18550.33 |
| P70 | 214.4 | 1003.76 |
| P71 |  | 86.67 |
| P72 | 0.0035 | 0.0066 |
| P73 | 0.025 | 0.07681 |
| P74 | 0.0002 | 0.00036 |
| P75 | 0.078 | 0.0021 |
| P76 | 0.0000493 | 0.00163 |
| P77 | 0.00489 | 0.01256 |
| P78 | 0.001 | 0.00216 |
| P79 |  | 3.398 |
| P80 |  | 173.20 |
| P81 | 1.8 | 8.834 |
| P82 | 0.007 | 0.01871 |
| P83 |  | 161.89 |
| P84 | 0.003 | 0.00731 |
| P85 | 20 | 127.85 |
| P86 | 380 | 1897.50 |
| P87 |  | 125.55 |
| P88 | 15 | 92.9 |

**Table S6.** Short peptide hits from the machine learning model pipeline.

| **Name** | **Sequence** |
| --- | --- |
| T1 | EGSGS |
| T2 | ARNDP |
| T3 | YETDL |
| T4 | YYEMD |
| T5 | CEGSD |
| T6 | GSGGSG |
| T7 | YEDGRL |
| T8 | QKTVEG |
| T9 | KQPSSG |
| T10 | AKEYND |
| T11 | RAQMDL |
| T12 | VQVQSA |
| T13 | SGSGDN |
| T14 | DPESGG |
| T15 | DASSRAP |
| T16 | EVSNRDS |
| T17 | MRAEVQS |
| T18 | KASGGPE |
| T19 | KGSGDVEG |
| T20 | TELKTEYN |
| T21 | FEEKEESS |
| T22 | DPPDNSNR |
| T23 | KVQYYTNE |
| T24 | YEGTDPLDS |
| T25 | TKEEGSRGSS |
| T26 | MEAVVQSEIQM |
| T27 | VQIYEEARKFS |
| T28 | EEEEEEEEEEEG |
| T29 | MEEGPSDPGSRS |
| T30 | GSSEGSSDFGGD |
| T31 | AEYETVQNSFNQ |
| T32 | GEKPDEFESGSP |
| T33 | IYRFEPSKFIGE |
| T34 | EYEEVEASPEKET |
| T35 | FEENENDDGGGGD |
| T36 | QETEEQMELADKA |
| T37 | DSSDSSDSNSSSDS |
| T38 | FQEEQQQTEDELQK |
| T39 | ACENEDFEGIPGEA |
| T40 | VQGSDQSDSANVQR |
| T41 | NDDEDPKSHRDPSN |
| T42 | RGNNDIGSGFNDDP |
| T43 | HGEGTFTSDLSKQM |
| T44 | GNTRTAESGDEDFF |
| T45 | SGEGSFQPSQQNPQ |
| T46 | SSGSVGESSSKGPR |
| T47 | GGDSRDPSDKSDGG |
| T48 | GIGPKFQHSGGEPP |
| T49 | QGNRKTTKEGSNDL |
| T50 | CASSGRGDDNQPQH |
| T51 | EEYGPEEDGGEESG |
| T52 | EPTTEDLYFQSDND |
| T53 | TESTLNTAIPGDPR |
| T54 | RYEVRAELPGVDPD |
| T55 | NRLVQNPPKKFSGE |
| T56 | ARATAETDATANRG |
| T57 | FEFEFEPGGGRGDS |
| T58 | GSEGSEGSEGSEGSE |
| T59 | ADNEGGDQPNEKPTE |

**Table S7.** Features selected by the SVM-Linear with RFE model, and their relative importance (weights) used in the classification model. The distribution of the features between 88 positive peptides and 21 hits was compared using Mann-Whitney U-test. The features are grouped into two blocks, based on a *p*-value of 0.05.

| **Features** | **Weights** | **p value (Positive vs Hits)** |
| --- | --- | --- |
| M | −2.11915616 | 0.068102351 |
| FE | 2.02462194 | 0.092112917 |
| FQ | 1.822695793 | 0.381796192 |
| SS | 1.778301801 | 0.069724704 |
| VQ | 1.707530239 | 0.429001883 |
| NR | 1.691099674 | 0.155903629 |
| TA | 1.656517936 | 0.096863226 |
| GA | −1.572397513 | 0.130251007 |
| GF | 1.562609164 | 0.466128639 |
| LF | −1.416414114 | 0.641758089 |
| _ChargeT12 | 1.410457016 | 0.428116505 |
| PG | 1.405464291 | 0.663727963 |
| IsoelectricPoint | −1.379260773 | 0.838773747 |
| _SecondaryStrD3100 | −1.345312744 | 0.276930314 |
| HR | 1.287933376 | 0.485106598 |
| NH | −1.22456886 | 0.498078928 |
| LD | −1.191514056 | 0.1088556 |
| WP | −1.155183359 | 1 |
| _ChargeT23 | 1.153226017 | 0.814687423 |
| KY | −1.152192578 | 0.327650367 |
| P | 1.1489593 | 0.740761114 |
| NF | −1.013993862 | 0.270397936 |
| GM | 1.009877196 | 0.498078928 |
| CI | −1.002895383 | 0.270364149 |
| SG | 0.996783331 | 0.454226212 |
| QM | 0.985901531 | 0.940864816 |
| _PolarizabilityD1050 | 0.97658651 | 0.334780155 |
| VA | −0.971072213 | 0.108852331 |
| MG | 0.969016614 | 0.400681038 |
| SQ | −0.966966243 | 0.779275233 |
| WV | −0.960493387 | 0.641758089 |
| LV | 0.948222389 | 0.080626854 |
| _PolarityD2050 | 0.875434718 | 0.204607336 |
| DR | −0.871623309 | 0.498078928 |
| YE | 0.860623162 | 0.746160355 |
| LR | 0.859662184 | 0.07597687 |
| PY | −0.845219798 | 0.641758089 |
| PN | −0.780243365 | 0.327650367 |
| PS | 0.74750734 | 0.722019797 |
| GG | 0.745039057 | 0.079675679 |
| _ChargeT13 | −0.73117318 | 0.262660756 |
| _ChargeD1001 | 0.692096528 | 0.222923747 |
| KF | 0.681387837 | 0.358918933 |
| EG | 0.676804995 | 0.694364304 |
| GY | −0.668802302 | 0.224386047 |
| AQ | −0.651579902 | 0.224386047 |
| _SolventAccessibilityC2 | 0.644295316 | 0.055529419 |
| _SecondaryStrC1 | −0.63634707 | 0.362411143 |
| _ChargeC2 | −0.618343251 | 0.508605116 |
| NQ | 0.615391799 | 0.30271378 |
| IG | 0.600417936 | 0.155917695 |
| W | −0.580516189 | 0.09096764 |
| NI | −0.577338361 | 0.498078928 |
| LK | −0.552081335 | 0.327650367 |
| YG | 0.526259001 | 0.155922384 |
| _ChargeC3 | 0.517447293 | 0.655735507 |
| RE | −0.499889429 | 0.27038949 |
| AF | −0.482824878 | 0.400681038 |
| RA | 0.47228752 | 0.443429668 |
| GP | 0.472171687 | 0.938436821 |
| WY | 0.461263366 | 0.498078928 |
| IK | −0.454324938 | 1 |
| TF | 0.45313904 | 0.220465999 |
| ND | 0.449345569 | 0.983351419 |
| GS | 0.412362786 | 0.413641442 |
| NP | −0.411075856 | 0.503342836 |
| _NormalizedVDWVC2 | −0.409912623 | 0.346457668 |
| LM | −0.398150729 | 0.641758089 |
| VI | −0.356181522 | 0.641758089 |
| LA | −0.347175939 | 0.327650367 |
| TN | −0.345850881 | 0.641758089 |
| TH | −0.33532355 | 1 |
| TK | 0.304370013 | 0.347071654 |
| LN | 0.298301805 | 0.224379174 |
| GE | −0.245454009 | 0.38009882 |
| YY | 0.192050889 | 0.224386047 |
| MP | 0.178887469 | 0.400681038 |
| KW | 0.176724131 | 0.327650367 |
| TI | −0.16845449 | 0.498078928 |
| LL | −0.15600722 | 0.270397936 |
| MQ | −0.131443495 | 1 |
| AS | 0.12118231 | 0.220468981 |
| MW | −0.118057549 | 1 |
| CS | −0.116633403 | 0.155922384 |
| DN | 0.093236846 | 0.096544141 |
| HS | −0.024086737 | 0.940864816 |
| AH | −0.003888531 | 0.498078928 |
| L | −2.064735702 | 3.31E−05 |
| CE | 1.717793408 | 0.049335189 |
| EY | 1.59982824 | 0.013080233 |
| _PolarizabilityD2075 | 1.429704395 | 1.70E−05 |
| _SecondaryStrD3050 | 1.428495226 | 0.011135344 |
| _NormalizedVDWVD2001 | −1.389250023 | 0.00018969 |
| SL | 1.357786754 | 0.043960408 |
| _SecondaryStrD2100 | −1.064588148 | 3.07E−05 |
| CL | 1.053744332 | 0.030304267 |
| _HydrophobicityT12 | 1.05182319 | 0.024582279 |
| _NormalizedVDWVD2075 | 0.982338351 | 2.07E−05 |
| _SecondaryStrD1100 | 0.975286204 | 0.002388777 |
| DF | 0.969705075 | 0.002556969 |
| _SecondaryStrC3 | 0.956335981 | 0.034213062 |
| VP | 0.937311738 | 0.036515141 |
| DG | −0.868173678 | 0.006266898 |
| SE | −0.835342555 | 0.030304267 |
| _ChargeD3100 | 0.647752509 | 0.01245346 |
| _HydrophobicityC3 | −0.620381164 | 0.000170426 |
| I | −0.577990388 | 0.00456863 |
| Weight | 0.539061979 | 3.44E−07 |
| A | −0.534332182 | 0.04098655 |
| AI | 0.503308816 | 0.043964504 |
| _PolarizabilityD2025 | 0.457510568 | 0.017351687 |
| _PolarizabilityD3025 | 0.384433783 | 0.00164178 |
| _NormalizedVDWVD3025 | 0.384433783 | 0.00164178 |
| AD | 0.328507843 | 0.017099044 |
| _HydrophobicityD3050 | 0.322302692 | 0.01060986 |
| VC | 0.242416708 | 0.007699247 |
| _ChargeD3001 | −0.129682459 | 8.73E−05 |
| SeqLength | 0.124473616 | 9.31E−07 |
| DY | −0.087165235 | 0.03652775 |
| PE | 0.054061206 | 0.001770935 |
